# A Survey of Optimization Methods for Independent Vector Analysis in Audio Source Separation

**DOI:** 10.3390/s23010493

**Published:** 2023-01-02

**Authors:** Ruiming Guo, Zhongqiang Luo, Mingchun Li

**Affiliations:** 1 School of Automation and Information Engineering, Sichuan University of Science and Engineering, Yibin 644000, China; 2 Artificial Intelligence Key Laboratory of Sichuan Province, Sichuan University of Science and Engineering, Yibin 644000, China

**Keywords:** blind source separation (BSS), independent component analysis (ICA), independent vector analysis (IVA), optimization update rule

## Abstract

With the advent of the era of big data information, artificial intelligence (AI) methods have become extremely promising and attractive. It has become extremely important to extract useful signals by decomposing various mixed signals through blind source separation (BSS). BSS has been proven to have prominent applications in multichannel audio processing. For multichannel speech signals, independent component analysis (ICA) requires a certain statistical independence of source signals and other conditions to allow blind separation. independent vector analysis (IVA) is an extension of ICA for the simultaneous separation of multiple parallel mixed signals. IVA solves the problem of arrangement ambiguity caused by independent component analysis by exploiting the dependencies between source signal components and plays a crucial role in dealing with the problem of convolutional blind signal separation. So far, many researchers have made great contributions to the improvement of this algorithm by adopting different methods to optimize the update rules of the algorithm, accelerate the convergence speed of the algorithm, enhance the separation performance of the algorithm, and adapt to different application scenarios. This meaningful and attractive research work prompted us to conduct a comprehensive survey of this field. This paper briefly reviews the basic principles of the BSS problem, ICA, and IVA and focuses on the existing IVA-based optimization update rule techniques. Additionally, the experimental results show that the AuxIVA-IPA method has the best performance in the deterministic environment, followed by AuxIVA-IP2, and the OverIVA-IP2 has the best performance in the overdetermined environment. The performance of the IVA-NG method is not very optimistic in all environments.

## 1. Introduction

With the advent of the era of big data information, people’s access to information has become more and more abundant. However, we usually only obtain the mixed information collected from the receiver, and the whole mixed information needs to be separated or extracted from the latent signals. The subsequent problem is how to effectively obtain useful signals from the received signals, which leads to the technology related to blind source separation (BSS) [1].

The theory of BSS can be traced back to the cocktail party problem, which has attracted much attention for decades. The cocktail party problem is when you are at a cocktail party and there are all kinds of people chatting around, but you can only concentrate on one of the discussions, or focus on the conversation of one of the people. BSS theory refers to observing the mixed signals of different sources and using these mixed signals to restore the original signal, and the prior information of the source signal and its mixed signal is minimal. A large number of applications of BSS in communication, speech, and medical signal processing has received extensive attention in recent years [2]. It is of great significance to realize blind estimation, blind equalization, and adaptive signal processing through blind characteristics.

Independent component analysis [3,4,5] (ICA) is one of the most important methods first proposed to deal with BSS. This is a classic BSS technology based on statistical independence of source signals and is the mainstream technology of BSS. ICA requires that source signals be statistically independent of each other. It is an unsupervised, data-driven signal processing technique based on non-Gaussian maximization to separate time-invariant mixture signals in the time domain.

However, consider that in a real scenario, the signal is often mixed with reverberation in the form of convolution. However, ICA cannot separate the common form of convolution mixing. Moreover, the convolution mixed signal is processed in the time domain with high computational complexity and a huge amount of computation, and the convergence speed is slow, which greatly reduces the separation performance. Taking advantage of the properties of convolution mixing: the convolution in the time domain is equal to the product in the frequency domain, a frequency domain ICA [6,7] (FD-ICA) algorithm is proposed. The entire convolutional mixed signal is converted from the time domain to the frequency domain for separation by the short-time Fourier transform (STFT). Compared with the time-domain convolution operation, the frequency-domain product operation has the advantages of convenient calculation, small computational complexity, and fast convergence speed.

**Figure 1:**
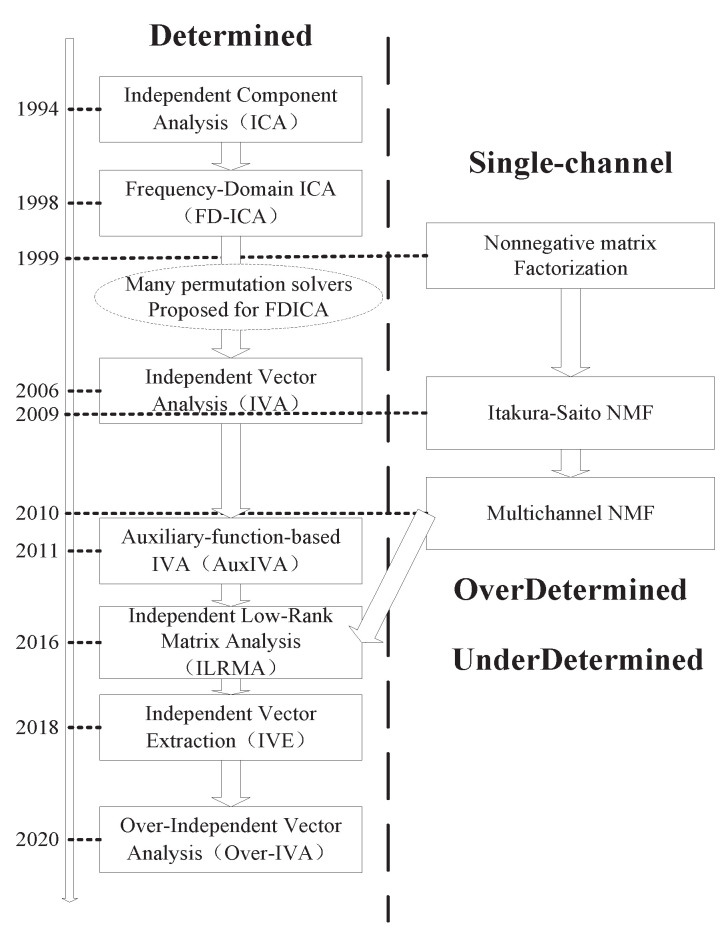
The history of IVA.

To solve the above-mentioned problems of ICA, the independent vector analysis (IVA) [8,9] algorithm is proposed. It generalizes ICA to multiple datasets by exploiting statistical dependencies across datasets, addressing some of the uncertainty in the output of signal separation. The method maintains the correlation of each source vector during the learning process while minimizing the correlation between different source vectors. Therefore, the permutation problem can be solved naturally without any pre- or post-processing during the learning process. Their entire development process is shown in Figure 1.

Since the IVA algorithm was proposed, it has been widely used, especially in the fields of speech signal [10,11], medical image [12,13,14], communication [15,16], and acoustic detection of unmanned aerial vehicles [17]. In addition, the relevant identification conditions and performance limit constraints of the IVA algorithm are also carried out [8]. In different application scenarios, the choice of the source prior model and the optimization update rule of the IVA algorithm will have different effects on the entire separation result. The source prior model that conforms to the scene will make the separation effect better, and the update rule determines the convergence accuracy and convergence speed, which plays a crucial role in the entire separation process.

**Figure 2:**
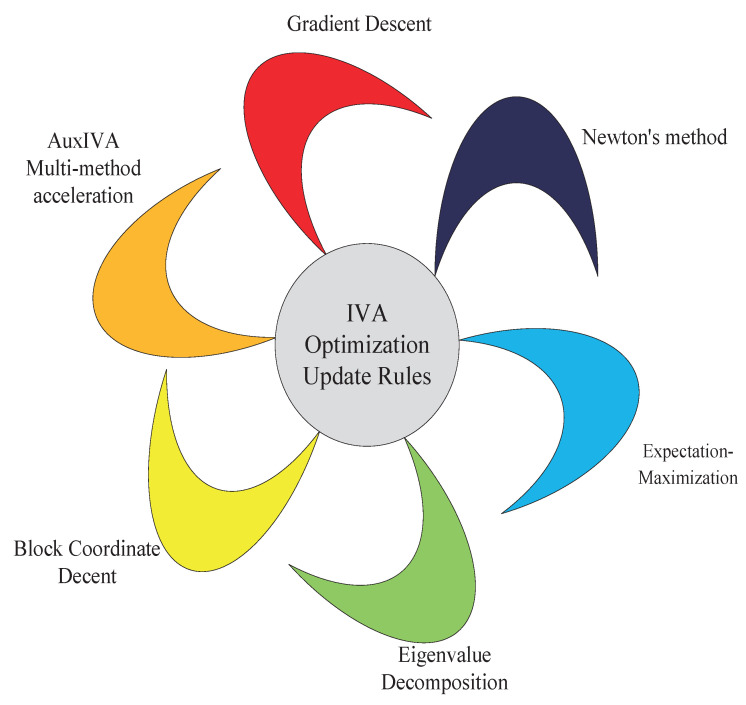
Optimizing update rule classification.

This paper mainly analyzes the state-of-the-art updated rules of the IVA algorithm, the mainstream technology of the BSS problem, such as gradient descent (GD), fast fixed point, auxiliary function, expectation maximization (EM), block coordinate descent (BCD), eigenvalue decomposition (EVD), and their derivation and mixed use are discussed comprehensively. Its classification is shown in Figure 2:

Each method has its unique effect on the convergence speed, convergence accuracy, and computational complexity of the entire calculation process of BSS. So far, although researchers have published and reported a great deal of work on the different update rules of the IVA, no comprehensive survey work has been conducted. In addition, the development of this work hopes that scholars who are interested in this field can get help and quickly become familiar with the current research status and future development trends in this field. Finally, the analysis of the optimization update rules in this paper is combined with the BSS problem, which will greatly promote the applications of the IVA algorithm to the BSS field.

The contributions of this paper are as follows. This paper reviews the existing kinds of literature on the application of a large number of publicly reported IVA algorithms in the field of BSS. The technical limitations and challenges of the IVA algorithms in dealing with the BSS problem under the existing optimization update rules are deeply analyzed. An in-depth analysis of the theory is provided to provide heuristic discussion and investigation guidance, as well as numerical experimental comparisons of optimization rules. The main contents of this article are as follows:

1. The theoretical principles of BSS for ICA and IVA are reviewed.
2. Analysis of the existing kinds of literature on IVA optimization update rule methods.
3. Numerical experiments are carried out to compare the existing optimization update rules.
4. Prospects are discussed for a series of research on the optimization and update rules and application fields of the IVA algorithm.

The remaining content of this paper is structured as follows. Section 2 discusses BSS and the principles underlying the theory and introduces their hybrid models and basic theories. Section 3 mainly analyzes the existing optimization update rule methods. Section 4 compares various methods numerically in overdetermined and deterministic conditions. Section 5 provides a summary and prospects.

## 2. The Principle of BSS

This section mainly introduces the two mainstream separation theories at this stage, analyzes the basic principles and models of separation, and summarizes the key principles of the two related algorithms. The specific content is as follows: In Section 2.1, the system model of BSS and related framework classifications are described, and then the basic separation algorithm principles related to BSS are introduced in Section 2.2 and Section 2.3.

### 2.1. Basic Model and Classification of BSS

The signal is usually mixed with the reverberation in a convolutional manner, and the mixing model is as follows,

(1) $$\begin{array}{cc} x_{m}\left[t\right]\; & =\sum_{n=1}^{N}a_{mn}\left[t\right]\times s_{n}\left[t\right]\\ \; & =\sum_{n=1}^{N}\sum_{\ell =0}^{L-1}a_{mn}\left[\ell \right]s_{n}\left[t-\ell \right],1\leq m\leq M,1\leq n\leq N. \end{array}$$

where $s_{n}\left[t\right]$ is the signal from source *n*, $x_{m}\left[t\right]$ is the *m*th observation value of *M* observations, $a_{mn}$ is a time-domain transfer function from the *n*th source to the *m*th observation, *t* represents time, *M* denotes the number of observation signals, and *N* denotes the number of source signals.

**Figure 3:**
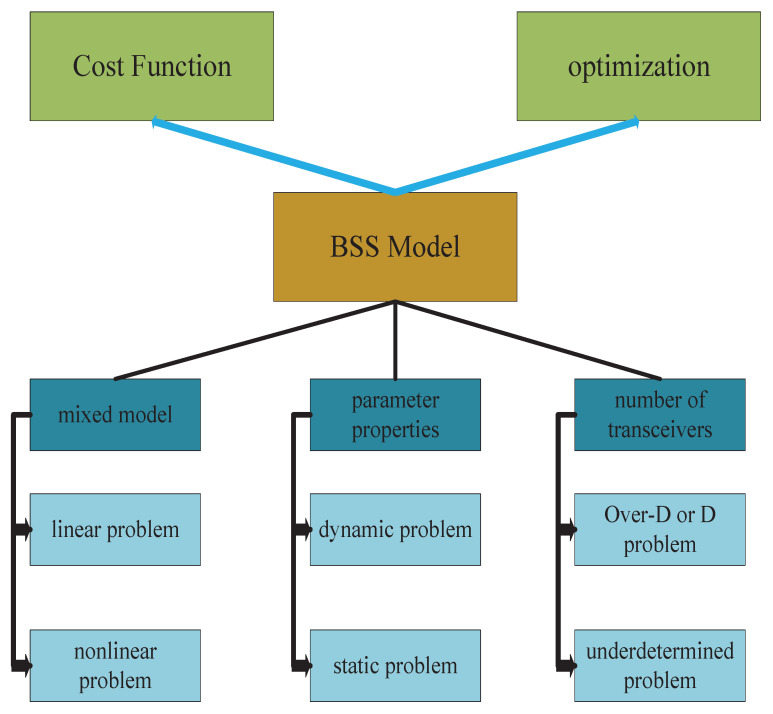
Basic elements of BSS problems.

The currently widely used ICA algorithm performs BSS by assuming that the source signals are statistically independent. The IVA algorithm is an extension of the ICA algorithm, which extends the ICA separation to multiple datasets for simultaneous decomposition. According to the relationship between the number of sensors that send and receive signals, the system models can be divided into three categories: determined models (M=N), underdetermined models (M>N), and overdetermined (M<N) models. It is a very meaningful and challenging problem to deal with underdetermined or overdetermined models that are more in line with real-life scenarios. In addition, different BSS problems can be described according to the mixture model and parameter characteristics, as shown in Figure 3.

Generally speaking, the model of the BSS algorithm consists of the cost function (objective function or loss function) and the optimization method. The cost function of BSS is constructed according to the characteristics of the restricted source and the separation criterion. The purpose of BSS is to find a suitable linear transformation matrix or separation matrix W by optimizing the cost function. So, in operation, the BSS separation process usually consists of two steps:

1. Estimate the separation matrix.
2. Use the estimated separation matrix to restore the source signal.

In the BSS problem, the cost function is always treated as an optimization problem, and the optimal solution is obtained by selecting the appropriate optimization algorithm in Figure 3 for optimization.

### 2.2. ICA and FD-ICA Algorithms

As an important theory of the BSS problem, the ICA algorithm relies on statistically independent criteria to construct the cost function. Then, according to the actual application scenario, an optimization algorithm is selected to optimize the cost function, and the source signal is separated or extracted from the linear mixed signal. The ICA algorithm is a characteristic unsupervised learning method that can directly estimate the mixture matrix and independent source components using only signal observations.

However, this method cannot separate the common form of convolution mixing, so the FD-ICA method is generally used for convolution mixing. This method transfers the whole convolution mixing separation to the frequency domain by STFT transform, and its model can be expressed as (2)

(2) $$x_{m}^{\left(k\right)}\left[z\right]=\sum_{n=1}^{N}a_{mn}^{\left(k\right)}s_{n}^{\left(k\right)}\left[z\right]$$

where $x_{m}^{\left(k\right)}\left[z\right]$, $s_{n}^{\left(k\right)}\left[z\right]$ denote the *m*th observation value of *M* observations, the *n*th source signal of *N* sources at the *k*th frequency bin, $a_{mn}^{\left(k\right)}$ denotes the mixing filter coefficients at the *k*th frequency bin, *z* denotes the time frame index, k=1,2,⋯,K, and *K* is the number of frequency bins. Compared with the time-domain convolution calculation, the complexity of the frequency-domain product calculation is significantly reduced. The complexity of frequency domain and time domain are M·logM and $M^{2}$, respectively.

In practical application scenarios, the ICA algorithm needs to satisfy subsequent assumptions to ensure effective and accurate signal source separation. The ICA model has three assumptions. First of all, the sources of data need to be independent of each other. The second is that the independent source components have a non-Gaussian probability distribution, that is, the higher-order statistics of the source are nonzero. Finally, the mixing matrix is a square invertible matrix. Usually, in most application scenarios, the first assumption is easier to satisfy. Because the sources in most scenarios come from different physical mechanisms, it is easy to create conditions that are independent of each other. While the second hypothesis reveals that the ICA algorithm cannot be applied to separate multiple Gaussian mixture signal scenarios, and therefore cannot separate the source signal from the Gaussian mixture. The square invertible matrix indicates that the number of source signals should be consistent with the observed quantity when mixing separation to ensure that the mixing matrix is invertible and can be used for source signal estimation.

However, the ICA algorithm and FD-ICA algorithm used in nonlinear functions have certain uncertainties, these uncertainties include the output signal sequence change, phase reversal and amplitude change. These uncertainties will directly lead to errors in signal separation and require subsequent processing for the fuzzy arrangement of separation results, thus increasing the overall separation complexity.

### 2.3. IVA Algorithm

**Figure 4:**
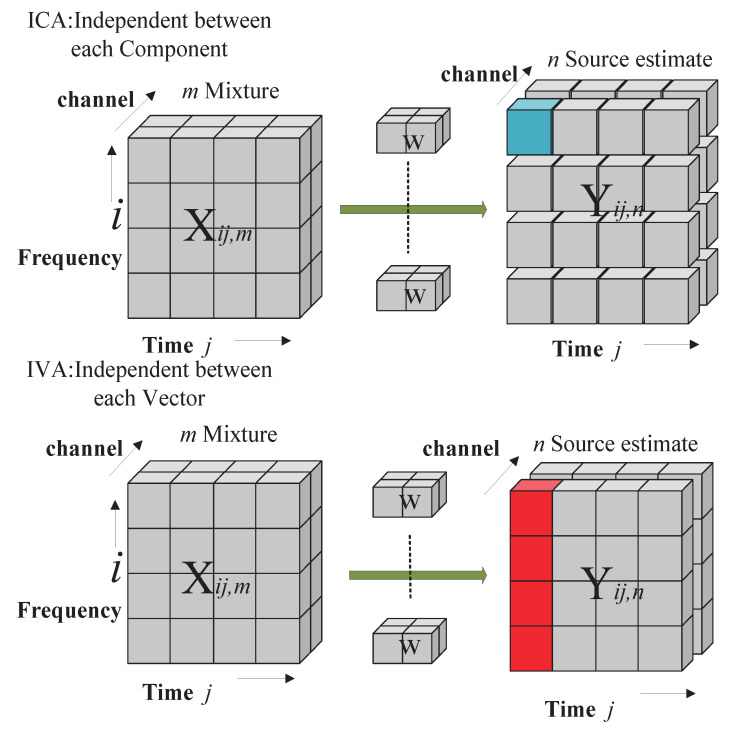
Models of ICA and IVA.

The IVA algorithm [8,9] is a new important theory that has been widely used in recent years to deal with BSS problems, and it is a frequency-domain method applied to convolution mixtures. Its model is composed of a set of standard ICA models, as shown in Figure 4.

The mixed model of noiseless frequency-domain convolution blind separation IVA can be represented by (2). Its separation model is expressed as (3)

(3) $$y_{n}^{\left(k\right)}\approx {\overset{\wedge }{y}}_{n}^{\left(k\right)}\left[z\right]=\sum_{m=1}^{M}w_{nm}^{\left(k\right)}x_{m}^{\left(k\right)}\left[z\right]$$

where $x_{m}^{\left(k\right)}\left[z\right]$, $s_{n}^{\left(k\right)}\left[z\right]$, and ${\overset{\wedge }{y}}_{n}^{\left(k\right)}\left[z\right]$ denote the *m*th observation value of *M* observations, the *n*th source signal, and *n*th estimated source of *N* sources at the *k*th frequency bin, and $y_{n}^{\left(k\right)}$ is the source signal. $a_{mn}^{\left(k\right)}$ and $w_{nm}^{\left(k\right)}$ are the mixing and unmixing filter coefficients at the *k*th frequency bin, respectively. Equations (2) and (3) can be simplified to vector representation:

(4) $$x^{\left(k\right)}\left[z\right]=A^{\left(k\right)}s^{\left(k\right)}\left[z\right],\;y^{\left(k\right)}\left[z\right]=W^{\left(k\right)}x^{\left(k\right)}\left[z\right]$$

where in (4) $x^{\left(k\right)}\left[z\right]={\left[x_{1}^{\left(k\right)}\left[z\right],x_{2}^{\left(k\right)}\left[z\right],\ldots \right]}^{T}$, $s^{\left(k\right)}\left[z\right]={\left[s_{1}^{\left(k\right)}\left[z\right],s_{2}^{\left(k\right)}\left[z\right],\ldots \right]}^{T}$, $y^{\left(k\right)}\left[z\right]=[y_{1}^{\left(k\right)}\left[z\right],$$y_{2}^{\left(k\right)}{\left[z\right],\ldots ]}^{T}$.

In this model, we assume that a linear mixed model exists in each dimension separately, and the underlying sources are independent of the other dimensions. Although it is an extension of the ICA algorithm, it differs from ICA in that the source signal is a random vector, not just a single variable. This means that the elements within the random component are closely related. Although the IVA algorithm is an extension of the ICA algorithm, the separation process of the IVA algorithm can be regarded as the separation of multiple ICA algorithm problems. However, instead of applying the ICA algorithm alone, the BSS problem is solved by defining a multivariate dependency and directly pushing it to the IVA algorithm. Three assumptions are proposed based on the ICA algorithm:

1. The elements in the source vector are independent of the elements of other source vectors.
2. In the source vector, there are dependencies among the elements.
3. The number of source signals should be less than or equal to the number of observed signals.

To be able to separate multivariate components from multivariate observations, we need to define contrast functions for multivariate random variables. By assuming dependencies among the elements of the source vector, we define the dependencies among vectors as the Kullback–Leibler (K-L) divergence between the product of the total joint probability of the vector and the marginal probability of the vector:

(5) $$\begin{array}{cc} I_{\mathrm{IVA}}\; & =\mathrm{KL}(f\left(y_{1},\cdots ,y_{N}\right)∥\prod_{n}g(y_{n}))\\ \; & =\sum_{n}E_{y_{n}}\log g\left(y_{n}\right)-2\sum_{K}\log|\det W^{\left(k\right)}|-\mathrm{const}. \end{array}$$

where $E_{y_{n}}$ denotes the expectation, $y_{n}^{\left(k\right)}={\left[y_{n}^{\left(k\right)},\ldots ,y_{n}^{\left(k\right)}\right]}^{T}$, $f(y_{1},\cdots ,y_{n})$ denotes the joint probability density function (PDF), and $\prod_{n}g(y_{n})$ denotes the product of approximate marginal probability density distribution functions, which is a nonlinear function. It should be noted that the random variables in the cost function are multivariate, and each source is also multivariate. In the separation process, the cost function is used to eliminate the dependency between the source vectors while retaining the connection between the source components. The source signals are separated by optimizing the cost function. Since the model preserves the elemental dependencies between each source signal, the separated source signals avoid the permutation problem. The IVA algorithm separates the entire dataset at the same time, which greatly improves the separation efficiency. Recently, an IVA algorithm combined with non-negative matrix factorization (NMF) for BSS independent low-rank matrix analysis (ILRMA) [18] was proposed. Using NMF as the source model of the IVA algorithm to capture the spectral structure solves the problem that IVA utilizes specific spectral structure features. specific spectral structure features.

**Figure 5:**
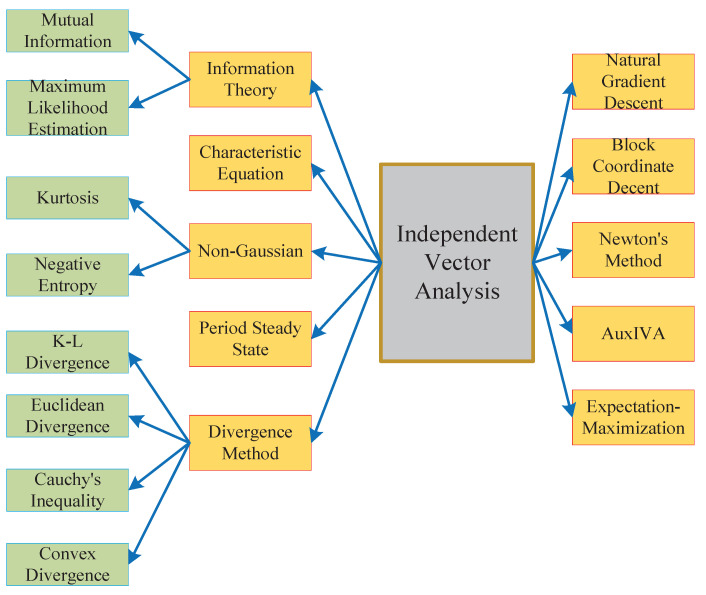
Basic components of IVA.

Based on the above description, the basic principle of BSS consists of two parts: cost function and optimization algorithm. The cost function of the IVA algorithm is usually constructed according to different statistically independent measures, including maximum likelihood estimation, mutual information, convex divergence, K-L divergence, and cumulant criterion. The optimization update rules include methods such as gradient descent, auxiliary function, and NI. The overall structure is shown in Figure 5.

This paper mainly discusses the existing IVA optimization and update rules comprehensively.

## 3. Optimizing IVA Algorithm—Optimizing Update Rules

The IVA algorithm resolves the permutation ambiguity of the ICA algorithm by exploiting the statistical dependencies between datasets. At the same time, the separation is extended to multiple datasets simultaneously, which greatly enhances the overall separation efficiency. Usually, the optimization update rule and the source prior model are the two most important factors when the IVA algorithm deals with different BSS problems.

The IVA algorithm solves the problem of arrangement ambiguity caused by the traditional algorithm by modeling the dependencies between the elements in the source component, that is, the source prior. Since the prior information provided by the source signal in different scenarios is different, choosing different source prior models in different scenarios will have a certain impact on the performance of the IVA algorithm. Especially in noisy environments, it is necessary to learn the parameters of the source prior and simultaneously realize the source components and denoising. Therefore, the selection of the source prior model determines whether the IVA algorithm can accurately capture the fine structure of the source signal, which plays a crucial role in the entire BSS process. In particular, the source prior model based on deep learning [19] and the source prior model based on deep neural network [20] are the focus of current source prior model research, and the source prior model is compared in detail in [20].

The selection of the source prior model is important for the overall separation process, but the choice of the algorithm update rule determines the separation efficiency. Usually, the IVA algorithm needs to optimize the separation matrix of all frequency points as a whole, and each iteration requires a relatively large amount of computation. When the separation matrix is initialized for the mixed signal, it will cause too many iterations and a long operation time, which will affect the separation performance. Moreover, when the initial value of the separation matrix is unreasonable, it is easy to fall into local convergence and it cannot be effectively separated. The most common is the update rule based on the step size, but the step size needs to be effectively selected to ensure the stability and convergence accuracy of the system. Therefore, the selection of update rules plays a decisive role in the stability and separation effectiveness of the separation process of the BSS problem. In the existing IVA algorithms, researchers have also developed many updated rules based on step size and nonstep size, as shown in Table 1.

This section surveys and discusses these valuable update rule methods. In this section, the relevant content is organized as follows. Section 3.1, Section 3.2 and Section 3.3 summarize the applications of GD, NI, and auxiliary function methods and their improved derivatives in BSS updating and describe the comparisons in the existing literature. The EM method is introduced in Section 3.4. The BCD method and its improvements are presented in Section 3.5. The EVD methods are introduced in Section 3.6.

### 3.1. Gradient Descent

GD [21] is one of the most primitive optimization algorithms. Gradient descent is a method that minimizes I by updating the model parameter in the opposite direction of the gradient of the objective function I. The learning rate η determines the size of the step size chosen to reach the local minimum, in other words, the descending hill along the slope of the surface produced by the objective function until a valley is reached. This is a separation method obtained by minimizing (5), a simple GD method is extrapolated as follows:

(6) $$\Delta W^{\left(k\right)}=-\frac{\partial I}{\partial W^{\left(k\right)}}$$

Its main variants are batch gradient (BG), stochastic gradient (SG), and natural gradient (NG). Among them, the NG algorithm [22,23] is an effective and one of the most commonly used algorithms to solve the problem of BSS. The main idea is to take the NG direction of the objective function I as the iterative direction so that the algorithm can quickly converge, so as to realize the separation of source signals. Additionally, it is proved that the best descent direction is not the “negative” regular gradient direction but the "negative" Riemann gradient. It was first proposed in [24,25], and its main idea is to multiply the scaling matrix $Q^{\left(k\right)}$ to modify the gradient in the original GD method to obtain faster convergence speed. As Equation (7):

(7) $$\Delta W^{\left(k\right)}=-\frac{\partial I}{\partial W^{\left(k\right)}}Q^{\left(k\right)}$$

The update for the separation matrix is:

(8) $$W^{\left(k\right)}\leftarrow W^{\left(k\right)}+\eta \Delta W^{\left(k\right)}$$

Both the conventional GD algorithm and its variants are inseparable from the choice of step size η when solving the objective function. The choice of step size will directly affect the convergence speed and accuracy. In order to speed up the convergence speed of the algorithm, many scholars have also optimized and improved the classical NG algorithm. In 2011, Liang et al. [26] proposed a control mechanism that considers the step size to obtain fast and stable convergence. In 2011, Zhang et al. [27] proposed an NG blind separation algorithm that directly estimates the score function through function approximation, which uses a linear combination of a set of orthogonal polynomials to approximate the score function, and its performance is measured by the mean squared error. An improved momentum term method was proposed in [28] which can speed up the algorithm’s convergence.

In 2018, Fu et al. [29] proposed a blind separation algorithm for IVA based on step-size adaptation. The algorithm initializes the separation matrix using the feature matrix joint approximate diagonalization algorithm and adaptively optimizes the step-size parameter. That is, to avoid local convergence, it can also significantly improve the convergence speed of the algorithm and further improve the separation performance. According to the relationship between the iteration step size and the estimated cost function change. In 2012, Wang et al. [30] proposed a variable-step-size IVA gradient algorithm based on the most block speed step-size descent. Additionally, according to the relationship between the iterative step size and the change in the separation matrix to be obtained, a variable-step-size IVA gradient algorithm based on the estimation function is proposed. In 2010, Kim [23] proposed a modified gradient and normalized IVA method with nonfully closed constraints. Gradient normalization improves the convergence speed, and nonholographically constrained gradients with lower computational complexity show better performance, while possessing simpler structures compared with other methods. In 2018, Koldovský et al. [31], based on the independent vector extraction (IVE) of the IVA algorithm, proposed an IVE algorithm with an adaptive step-size method in complex non-Gaussian scenarios to speed up convergence.

### 3.2. Fast Fixed Point Method

The fast fixed point method was derived by introducing Newton’s method. The iterative update rule based on fast fixed point [32] was first proposed to optimize the objective function of ICA. It provides a very simple algorithm, one that does not depend on any defined parameters and that quickly converges to the most accurate update rule the data allow.

When optimizing a negative entropy-based objective function, the easiest way is to use GD. Although the GD-based method has a good separation effect, it is relatively simple to use. The overall convergence speed of this method is slow and depends on a good choice of the learning rate sequence, i.e., the step size per iteration. Although various optimizations for the step-size factor were summarized in the previous section, GD methods rely on a suitable step size for separation.

Therefore, in practical applications, it is very important to make the entire convergence process faster and more reliable. Therefore, a fast fixed point iterative algorithm [33] is proposed to achieve this. In fixed point algorithms, the entire computation is performed in batch or block mode, i.e., a large number of data points are used in one step of the algorithm. The fast fixed point algorithm has very attractive convergence properties, and in experiments, it converges much faster than the commonly used GD method. At the same time, in environments where fast real-time adaptation is not required, this method is a good alternative to adaptive learning rules. In 1997, Hyvarinen [34] described a more heuristic derivation of it.

In 2000, Bingham et al. [35] proposed a FastICA algorithm capable of separating complex-valued linear mixed-source signals. The method shows good performance in the ICA algorithm. The same [36] generalized fast fixed point method to the IVA algorithm, which was developed based on the idea of FastICA and used to optimize the traditional IVA algorithm. Under this method, the update is expressed as:

(9) $$\begin{array}{c} w_{n}^{\left(k\right)}\leftarrow E[G^{\prime }\left(\sum_{k}|y_{n}^{\left(k\right)}{|}^{2}\right)\\ +|y_{n}^{\left(k\right)}{|}^{2}G^{\prime \prime }\left(\sum_{k}|y_{n}^{\left(k\right)}{|}^{2}\right)]w_{n}^{\left(k\right)}\\ -E[{\left(y_{n}^{\left(k\right)}\right)}^{*}G^{\prime }\left(\sum_{k}|y_{n}^{\left(k\right)}{|}^{2})x^{\left(k\right)}\right] \end{array}$$

where *E* denotes the expectation, G(·) denotes a nonlinear function, and

(10) $$G\left(\sum_{k}|y_{n}^{\left(k\right)}{|}^{2}\right)=-\log{\overset{\wedge }{g}}_{s_{n}}\left(y_{n}\right)$$

where $w_{n}^{\left(k\right)}={\left[w_{n}^{\left(k\right)},\ldots ,w_{n}^{\left(k\right)}\right]}^{T}$, $y_{n}={\left[y_{n}^{\left(1\right)},\ldots ,y_{n}^{\left(K\right)}\right]}^{T}$, and ${\overset{\wedge }{g}}_{s_{n}}\left(y_{n}\right)$ denotes the estimate of the source PDF, the source prior. ${\left(\cdot \right)}^{*}$ denotes the complex conjugate of (·). After the updated matrix W is obtained through the update rule, decorrelation needs to be performed to ensure orthogonality as follows:

(11) $$W^{\left[k\right]}\leftarrow {\left(W^{\left[k\right]}{\left(W^{\left[k\right]}\right)}^{H}\right)}^{-1/2}W^{\left[k\right]}$$

where ${\left(\cdot \right)}^{H}$ denotes the conjugate transpose of (·). To be able to directly apply Newton’s method to derive a fast algorithm for complex variables, a quadratic Taylor polynomial is introduced into the complex notation. Using this form of Taylor series expansion makes the derivation simpler and is useful for directly applying Newton’s method to objective functions of complex-valued variables. In 2000, Yan et al. [37] provided an independent equivalent.

Recently, in 2021, Koldovský et al. [38] proposed an extended fast dynamic independent vector analysis (FastDIVA) algorithm based on the FastICA and FastIVA static hybrid algorithms, used to blindly extract or separate one or more signal sources from a time-varying mixed signal. In a source-by-source separation mixture model that allows the desired source to move, the mixture is either in series or in parallel. The algorithm inherits the advantages of FastIVA, exhibits good performance in motion source separation, and exhibits superior convergence speed and ability to separate super-Gaussian and sub-Gaussian signals.

In 2021, Amor et al. [39] used FastDIVA for blind source extraction for mixture models with constant separation vector CSV. Additionally, it shows new potential and good separation performance in three environments: motion loudspeaker in a noisy environment, extraction of motion brain activity, and motion source. In 2021, Koldovský et al. [40] proposed a new dynamic IVA algorithm. It is based on a mixed model in which the source-of-interest (SOI)-related mixing parameters are time-varying, and the separation parameters are time-invariant. The Newton–Raphson method is used to optimize the objective function based on the quasi-likelihood method, then the iterative update is performed without imposing orthogonality constraints, and then orthogonality is performed. This algorithm is an optimization of the fast fixed point algorithm, which is better than the gradient algorithm and the auxiliary function method in performance.

### 3.3. Auxiliary Function

The update method based on the auxiliary function technology is also a method that does not include tuning parameters such as step size, which is an iterative algorithm with a convergence guarantee. This is a stable and fast update rule derived from the majorize-minimization principle [10,49]. Find its minimum by exploiting the convexity of the function. When the objective function f(θ) is difficult to optimize, and the optimization algorithm used cannot directly find the optimal solution to the objective function, an easy-to-optimize objective function g(θ) can be found instead. Then, the substitution function is solved, and the optimal solution of g(θ) is close to the optimal solution of f(θ). In each iteration, a new surrogate function for the next iteration is reconstructed from the solution. Then, the new substitute function is optimized and solved to obtain the objective function of the next iteration. After several iterations, the optimal solution that is closer and closer to the original objective function that can be obtained. It was first proposed in the literature [41] to accelerate the convergence speed of the ICA algorithm. This rule consists of two optional updates:

1. The update of the weighted covariance matrix (that is, the auxiliary function variable).
2. The update of the separation matrix ensures that the objective function decreases monotonically at each update and finally achieves convergence.

Equation (12) is the auxiliary function variable update:

(12) $$V_{n}=E_{n}\left[\frac{U^{\prime }\left(\right\|y_{n}{\|}_{2})}{\|y_{n}{\|}_{2}}x_{n}{\left(x_{n}\right)}^{H}\right]$$

Among them, $V_{n}$ denotes a covariance matrix of the observed signals, U(·) denotes a continuous and differentiable function of a real variable · satisfying, and $U^{\prime }\left(\cdot \right)$ usually takes the constant 1. ${\left\|\cdot \right\|}_{2}$ denotes the 2-norm of ·. Equation (13) is the update of the unmixing matrix:

(13) $$w_{n}^{\left(k\right)}=\frac{{\left[WV_{n}\right]}^{-1}e_{n}}{\sqrt{e_{n}^{T}\left(W_{n}^{-H}V_{n}^{-1}W_{n}^{-1}\right)e_{n}}}$$

In 2011, Nobutaka Ono [42] used the auxiliary function technique in the objective function of the IVA algorithm and similarly derived an efficient update rule suitable for the IVA algorithm, called AuxIVA. In 2012, Nobutaka Ono [43] proposed an AuxIVA algorithm based on a generalized Gaussian source model or a Gaussian source model with time-varying variance. In 2012 and 2013, Nobutaka Ono [44,45] proposed a faster algorithm that can update two separation vectors simultaneously by solving the generalized eigenvalue problem for the AuxIVA algorithm with two sources and two microphones. Compared with the one-by-one update method, this method has faster convergence speed and better performance. This pairwise update method is also applicable to the pairwise separation of vectors in the case of three or more sources [46]. In 2014, Taniguchi et al. [47] used the AuxIVA algorithm based on the auxiliary function method for online real-time blind speech separation. In experimental comparisons with commonly used real-time IVA algorithms, the proposed online algorithm achieves a higher signal-to-noise ratio without environment-sensitive tuning parameters such as step factor.

In 2021, Brendel et al. [48] further optimized the IVA algorithm based on auxiliary functions under the same computational cost. The convergence speed of the AuxIVA algorithm is enhanced by three methods:

1. Turn the differential term into a tuning parameter via the differential term in the NG approximation algorithm.
2. Approximate the differential term as a matrix using the quasi-Newton method.
3. Use the square iteration method to speed it up.

### 3.4. EM Method

In signal processing, a common problem is estimating the parameters of a probability distribution function. The situation is more complicated in many parameter estimation problems because the data needed to estimate the parameters are not directly accessible, or some data are missing. EM-based optimization algorithms are well-suited for solving this class of problems because the EM algorithm produces maximum likelihood (ML) estimates of the parameters when there is a many-to-one mapping from the underlying distribution to the distribution of the control observations, while taking additive noise into account. The EM algorithm overcomes the problem of unanalyzable solutions and has been widely used in statistics, signal processing, and machine learning [50].

The EM algorithm is an iterative optimization method [51] that is used to estimate some unknown parameters given measurement data. The solution is divided into two steps.

E-step: First assign an initial distribution to each hidden variable empirically, that is, assume distribution parameters. Then, according to the parameters of the distribution, the expectation of the hidden variables in each data tuple can be obtained, that is, the classification operation is performed. The posteriors of the source signal can be obtained by

(14) $$\begin{array}{cc} \log q\; & \left(x_{1}^{\left(k\right)},\ldots ,x_{N}^{\left(k\right)}|s_{1}^{\left(k\right)},\ldots ,s_{N}^{\left(k\right)}\right)\\ & \propto \log g(y_{1}^{\left(k\right)},\ldots ,y_{N}^{\left(k\right)}|x_{1}^{\left(k\right)},\ldots ,x_{N}^{\left(k\right)})\\ & +(\log g\left(x_{1}^{\left(k\right)}|s_{1}^{\left(k\right)}\right)+\ldots +\log g\left(x_{N}^{\left(k\right)}|s_{N}^{\left(k\right)}\right))+const. \end{array}$$

where ∝ denotes it is proportional to the previous term, and *q* denotes posterior probability.

M-step: Calculate the maximum likelihood value of the distribution parameter (vector) based on the classification result, and then in turn recalculate the expectation of the hidden variable for each data tuple based on this maximum likelihood value. The update rules for mixing matrices A are

(15) $$A^{\left(k\right)}=\left(\sum_{k}<y^{\left(k\right)}{\left(x^{\left(k\right)}\right)}^{T}{>}_{q}\right){\left(\sum_{k}<x^{\left(k\right)}{\left(x^{\left(k\right)}\right)}^{T}{>}_{q}\right)}^{-1}$$

where $<\cdot {>}_{q}$ denotes expectation over *q*.

Through the repetition of the above two steps, when the expectation of the hidden variable and the maximum likelihood value of the parameter tends to be stable, the entire iteration is completed.

In 2004 and 2008, Varadhan et al. [52,53] used the square iteration method in the EM algorithm to accelerate its convergence speed. In 2008, Lee et al. [54] deduced the expectation-maximization algorithm, and the algorithm was used in the updated iteration of the IVA algorithm. The EM algorithm could estimate the parameters of the separation matrix and the unknown source at the same time, showing a good separation performance. In 2010, Hao et al. [55] proposed a unified probabilistic framework for the IVA algorithm with the Gaussian mixture model as the source prior model; this flexible prior source enables the IVA algorithm to separate different types of signals, deduce different EM algorithms, and test three models: noiseless IVA, online IVA, and noise IVA. The EM algorithm can effectively estimate the unmixing matrix without sensor noise. In online IVA, an online EM algorithm is derived to track the motion of the source under nonstationary conditions. Noise IVA includes sensor noise and denoising combined with separation. An EM algorithm suitable for this model is proposed which can effectively estimate the model parameters and separate the source signal at the same time.

In 2019, Gu et al. [56] proposed a Gaussian mixture model IVA algorithm with time-varying parameters to accommodate temporal power fluctuations embedded in nonstationary speech signals, thus avoiding the pretraining process of the original Gaussian mixture model IVA (GMM-IVA) algorithm and using the corresponding improved EM algorithm to estimate the separation matrix and signal model. The experimental results confirm the effectiveness of the method in random initialization and the advantages in separation accuracy and convergence speed. In 2019, Rafique et al. [57] proposed a new IVA algorithm based on Student’s t-mixture model as a source before adapting to the statistical properties of different speech sources. At the same time, an efficient EM algorithm is derived which estimates the location parameters of the source prior matrix and the decomposition matrix together, thereby improving the separation performance of the IVA algorithm. In 2020, Tang et al. [58] proposed a complex generalized Gaussian mixture distribution with weighted variance to capture the non-Gaussian and nonstationary properties of speech signals to flexibly characterize real speech signals. At the same time, the optimization rules based on the EM method are used to estimate and update the mixing parameters.

### 3.5. BCD Method

Coordinate descent (CD) is a nongradient optimization algorithm. The algorithm does not need to calculate the gradient of the objective function and performs a linear search along a single dimension at a time. When a minimum value of the current dimension is obtained, different dimension directions are used repeatedly, and the optimal solution is finally converged. However, this algorithm is only suitable for smooth functions. When nonsmooth functions are used, they may fall into a nonstagnant point and fail to converge. In 2015, Wright [59] proposed block coordinate descent (BCD), a generalization of the coordinate descent algorithm. It decomposes the original problem into multiple subproblems by simultaneously optimizing a subset of variables. The order of updates during the descent can be deterministic or random. This algorithm is mainly used to solve the nonconvex function, of which the objective function’s global optimal value is difficult to obtain.

Among them, the BCD algorithm has developed two methods with closed update formula for the BSS IVA algorithm’s [60] IP and ISS methods.

#### 3.5.1. Iterative Projection

**Figure 6:**
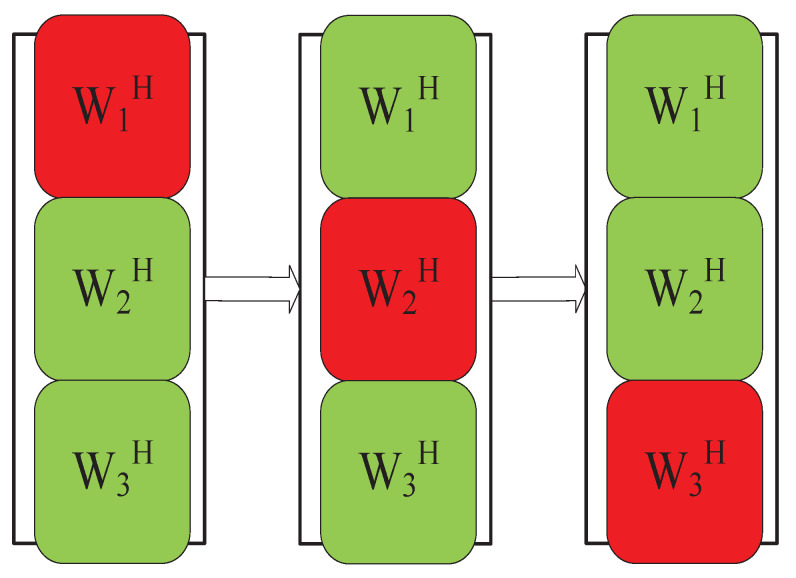
IP update method.

The IVA algorithm based on iterative projection was first introduced in the AuxIVA [42] algorithm. Its update rule is similar to (13). Figure 6 shows that the algorithm alternately updates each row vector of the separation matrix during each iteration of block coordinate descent, where red denotes the vector to be updated and green denotes the mixed vector.

**Figure 7:**
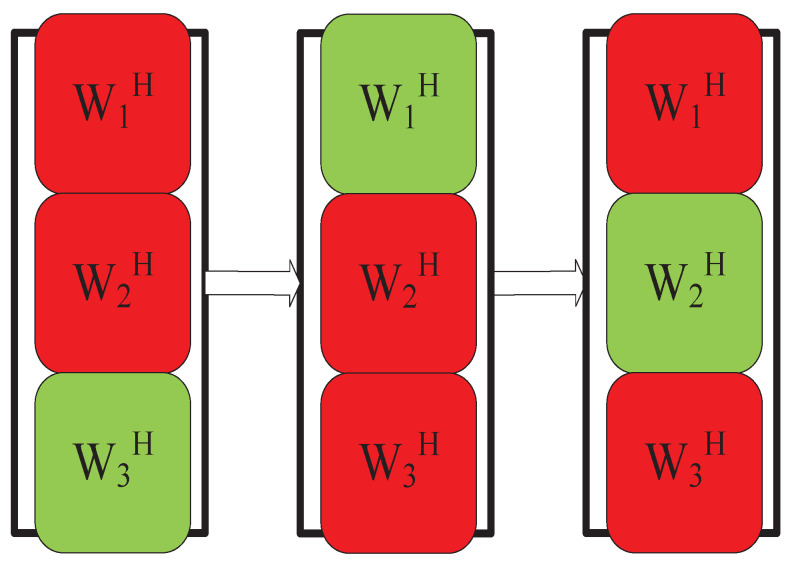
IP-2 update method.

This update rule is derived by solving a quadratic system of equations obtained by differentiating the cost function concerning the separation vector. In 2004, Dégerine et al. [61] also proposed a similar scheme in the context of semiblind Gaussian source components. In 2016, Kitamura et al. [62] used the IP algorithm in a BSS algorithm combining IVA and NMF, which provided good convergence speed and separation effect. In 2018, Yatabe et al. [63] proposed an alternative to the AuxIVA-IP algorithm based on proximal splitting. In 2021, Nakashima et al. [64] optimized it based on IP and extended each row vector of the separation matrix to update one by one to two rows of the separation matrix per update, resulting in a faster IP-2, as shown in Figure 7:

In 2020, Ikeshita et al. [65] deduced IP-1 and IP-2 and used these two update rules to accelerate the OverIVA algorithm, forming the OverIVA-IP and OverIVA-IP2 update rules. In 2021, Scheibler [66] proposed an iterative projection with adjustment (IPA) and a Newton conjugate gradient (NCG) to solve the hybrid exact-approximate diagonalization (HEAD) problem. IPA adopts a multiplicative update form, that is, the current separation matrix is multiplied by the rank 2 perturbation of the identity matrix. This method performs joint updates to the unmixing filters and additional rank-one updates to the remainder of the unmixing matrix. Simply put, the IPA optimization rule is a combination of IP and ISS methods. Updating one row and one column of the matrix in each update, performing IP- and ISS-style updates jointly, outperforms the IP and ISS methods.

#### 3.5.2. Iterative Source Steering

**Figure 8:**
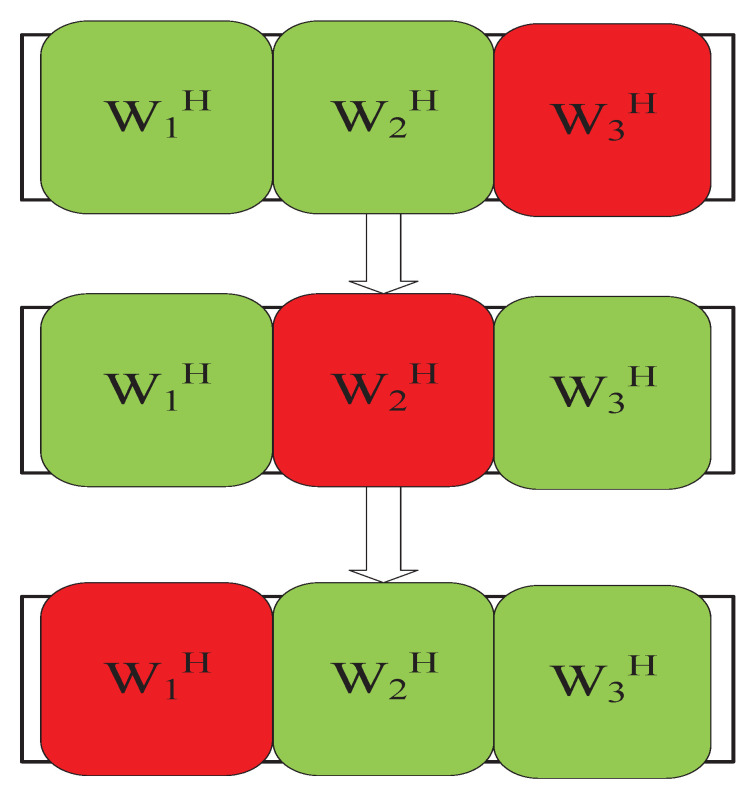
ISS update method.

ISS [67] is an alternative to IP. Although IP has the advantages of good performance and fast convergence speed, in the iterative update process, it needs to recalculate a covariance matrix and invert for each source and each iteration. This greatly increases the overall complexity of the algorithm. The complexity of the algorithm is three times the number of microphones used. In addition to that, inverting a matrix is an inherently dangerous operation that can lead to unstable convergence when iterating. On this basis, the proposed ISS algorithm can effectively reduce the computational cost and complexity brought by the IP algorithm. ISS can also minimize the same cost function as the AuxIVA algorithm. Figure 8 shows that the algorithm considers a series of rank-1 updates to the separation matrix itself throughout the separation process, i.e., updating one column of the separation matrix, rather than updating one separation matrix at a time. The update method is as follows:

(16) $$W^{\left(k\right)}\leftarrow W^{\left(k\right)}-v_{n}^{\left(k\right)}{{(w}_{n}^{\left(k\right)})}^{H}$$

where $w_{n}^{\left(k\right)}={\left[w_{n}^{\left(1\right)},\ldots ,w_{n}^{\left(K\right)}\right]}^{T}$. This update method is inverse, and the complexity of each iteration is only quadratic times the number of microphones.

This update rule, which does not require matrix inversion, is used in a new method for joint deredundancy and BSS [68]. This is a method based on an ILRMA framework, which combines the advantages of no inversion and low complexity of the ISS algorithm to achieve efficient BSS. In 2021, Du et al. [69] proposed a computationally efficient optimization algorithm for BSS of overdetermined mixtures, an improved ISS algorithm for OverIVA algorithm, namely OverIVA-ISS. The algorithm combines the technology in OverIVA-IP with the technology in AuxIVA-ISS, which is more computationally efficient than the OverIVA-IP algorithm and can guarantee convergence. Additionally, the computational complexity is reduced from $O(M^{2})$ to O(MN).

**Figure 9:**
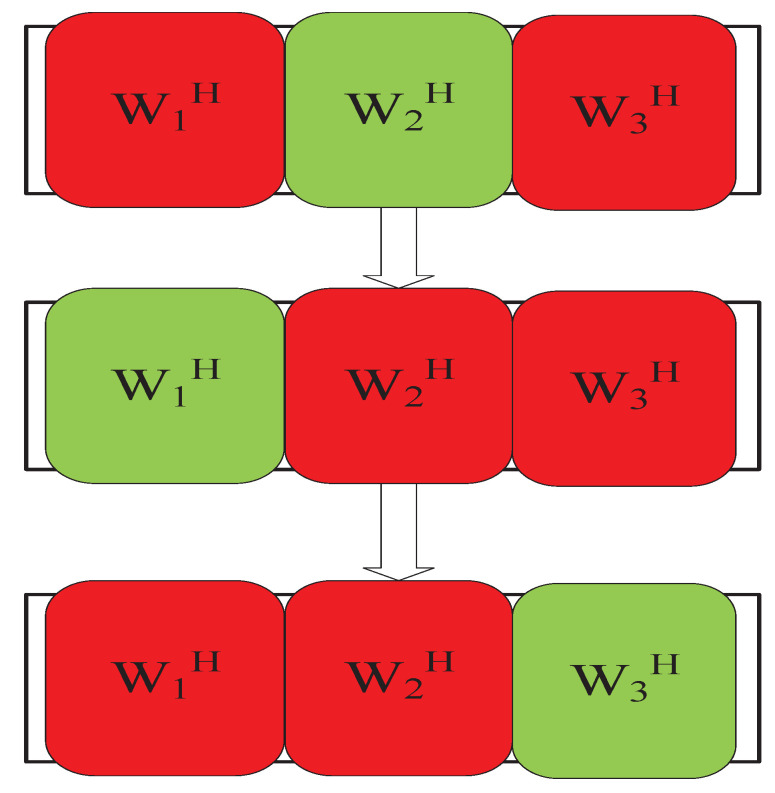
ISS-2 update method.

The overall performance of the ISS algorithm is better than the IP algorithm but inferior to the IP-2 algorithm. Therefore, an ISS-2 algorithm is proposed. In 2022, Ikeshita et al. [70] extended the ISS algorithm to ISS-2; Figure 9 shows that the latter can update two rows of the separation matrix at each iteration.

At the same time, the advantage of the smaller time complexity of the ISS algorithm is maintained, and the separation performance is comparable to IP-2.

### 3.6. EVD Method

The EVD method is to find the most similar matrix to the original matrix. The optimization update rule based on EVD can be expressed as:

(17) $$w^{\left[k\right]}\leftarrow \frac{w^{\left[k\right]}}{{\|w^{\left[k\right]}\|}_{2}}$$

and

(18) $$w^{\left(k\right)}=\frac{1}{\sqrt{\lambda_{M}^{\left(k\right)}}}u_{M}^{\left(k\right)}$$

where $\lambda_{M}$ and $u_{M}$ denote the smallest eigenvalue and eigenvector, respectively.

The IVA algorithm based on the EVD update rule was proposed in [11] for a fast independent vector extraction (FIVE) algorithm. By comparing with the OverIVA and AuxIVA algorithms experimentally, the proposed algorithm can obtain the optimal solution with only a few iterations and is far superior to other algorithms in terms of convergence performance. In 2021, Brendel et al. [71] extended the update rule of eigenvalue decomposition to an IVA source extraction algorithm with SOI mechanism. The proposed update rule achieves fast convergence at lower computational cost and outperforms the IP update rule in performance.

### 3.7. Summary

Regarding the above optimization update rules, the NG method needs to set the step size and other tuning parameters for iteration, and the convergence is slow. When the tuning parameters are not appropriate, it will cause convergence failure. Newton’s method is faster to converge but computationally more complex. The method based on the auxiliary function can effectively estimate the source signal by constructing the auxiliary function to replace the intractable objective function. At the same time, the combination acceleration can be performed by the other methods mentioned above, but this method has a large amount of calculation and high complexity. The EM method can estimate parameters more easily to achieve convergence and can deal with scenarios where parameter estimation is complicated or impossible. The BCD method mainly deals with convex functions that are difficult to obtain the global optimum. At the same time, this method can be used in combination with other algorithms and is one of the most widely used methods at present. May fail to converge when dealing with nonsmooth functions. The EVD method unmixes and updates the mixing matrix through eigenvalue decomposition, the update speed is faster, and it is mostly used for the extraction of a single source. All methods other than NG do not require tuning parameters such as step size.

## 4. Optimizing the Performance Comparison of Update Rules

### 4.1. Frequency Domain Convolution Blind Separation

Typically, a microphone in a reverberant environment records a real-valued convolution mix of all sources in the scene, as described in Function (1). The time-domain signal is divided into frames and then multiplied by a window function, and the time-domain signal is converted into a frequency-domain representation through STFT, such as in Function (2), which effectively reduces the amount of calculation and complexity. In this experimental environment, Gaussian white noise is added to (1), and its model is expressed as (19):

(19) $$x_{m}\left[t\right]=\sum_{n-1}^{M}\sum_{\ell =0}^{L-1}a_{mn}\left[\ell \right]s_{n}\left[t-\ell \right]+b_{m}\left[t\right]$$

where $b_{m}\left[t\right]$ is the uncorrelated microphone noise signal.

### 4.2. Experimental Environment Settings

**Figure 10:**
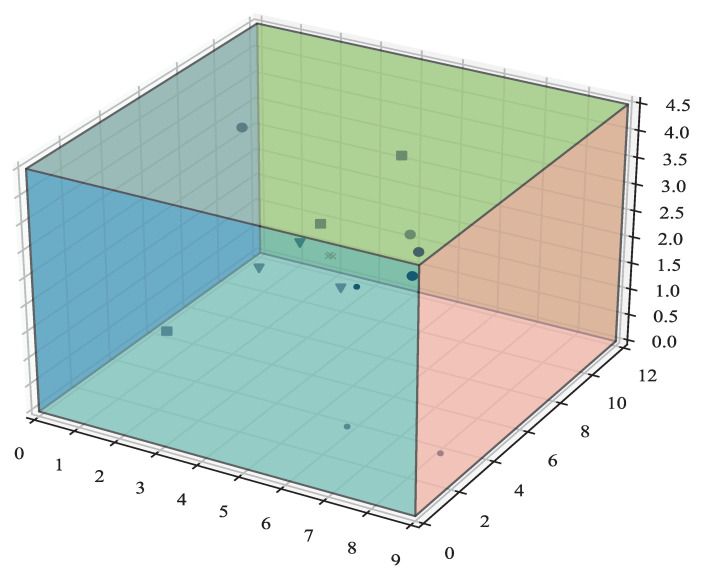
Simulation of a 3D matrix room.

By using the pyroroomacoustics Python package to simulate 1000 random 3D matrix rooms, the source and interfering signals are randomly distributed in the 3D room, as shown in Figure 10:

This three-dimensional matrix room has wall lengths of 6 m and 10 m and ceiling heights from 2.8 to 4.5 m. The simulated reverberation time is uniformly sampled between approximately 60 ms and 450 ms. The source and microphone array are randomly placed at least 50 cm away from the wall, and the height is between 1 and 2 m. The array is circular and regular, and the number of microphones that can be selected is between 3 and 10, with a radius of 10 cm between adjacent microphones. All sound sources are located farther from the array than the critical distance of the room, where the direct sound and reverberation energy are equal. This distance can be calculated by Equation (20):

(20) $$d=0.057\sqrt{V/T_{60}}$$

where *V* represents the volume of this room. At the same time, the SNR of each microphone is defined as:

(21) $${SNR}_{mic}=\frac{E[\|x_{m}\left[\ell \right]-b_{m}\left[\ell \right]{\|}^{2}]}{E[\|b_{m}\left[\ell \right]{\|}^{2}]}$$

Obtain a specified SNR at any reference microphone by adding uncorrelated Gaussian noise $b_{m}[\ell ]$ to the microphone output. In the comparison experiment, the first microphone was selected as a reference, and its SNR value was fixed. SNR values of 5 dB, 15 dB, and 25 dB were investigated. Experiments under different signal-to-noise ratios can directly reflect the impact of noise on the algorithm performance in blind source separation. If the separation performance is different, the impact of noise is large; otherwise, the impact of noise is small. Simulations were performed at 16 kHz using speech signals from the CUM Arctic corpus, using a 4096 Hamming window with STFT overlapping 3/4. Through the separation of convolutional mixed speech signals, we comprehensively compare the performance of various IVA optimization update rule algorithms such as AuxIVA-IP, AuxIVA-IP2, AuxIVA-ISS, FastIVA, NGIVA, and OverIVA-IP, reviewed in the previous chapter.

### 4.3. Experimental Simulation Results

In simulation experiments, the multivariate Laplacian source prior model is used in various IVA methods for performance evaluation. In the process of the BSS experiments, two cases of determined model 3 × 3 and overdetermined model 4 × 3 were considered. The experimental configuration is carried out under the same number of interference signals and target signal environment.

**Figure 11:**
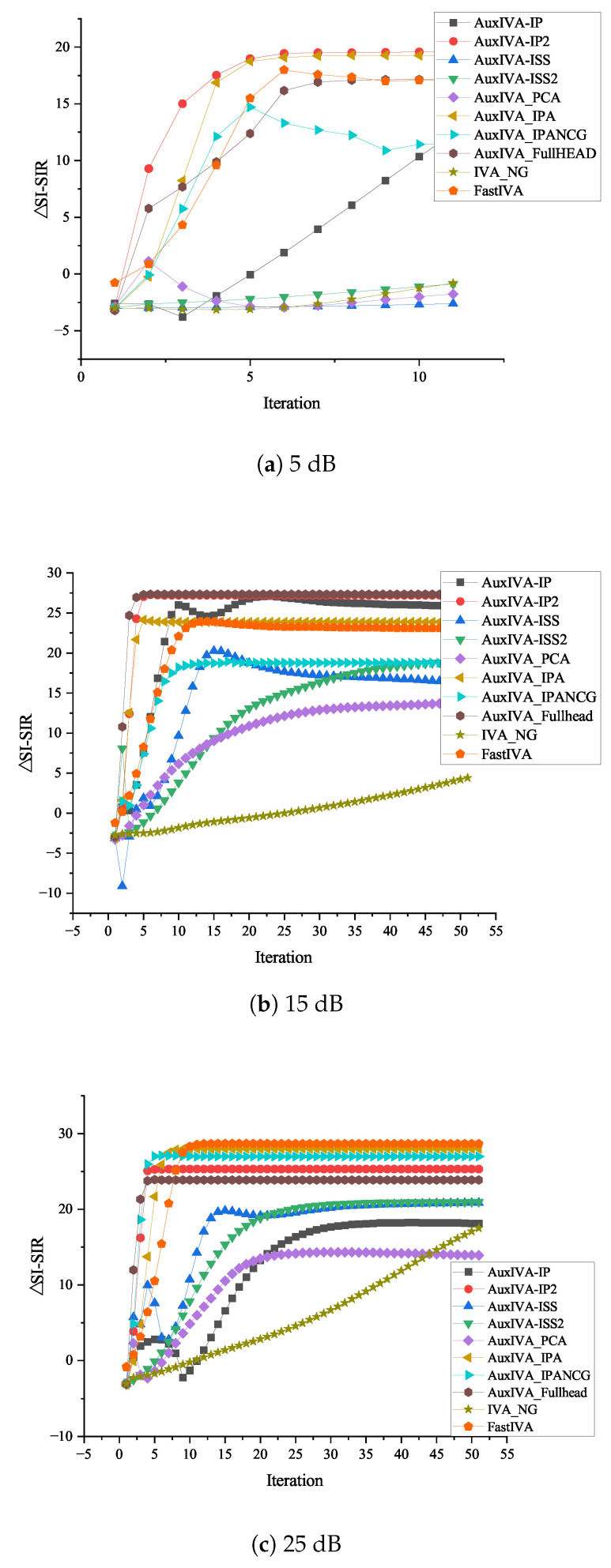
Performance comparison under different SNRs in the 3 × 3 determined case.

**Figure 12:**
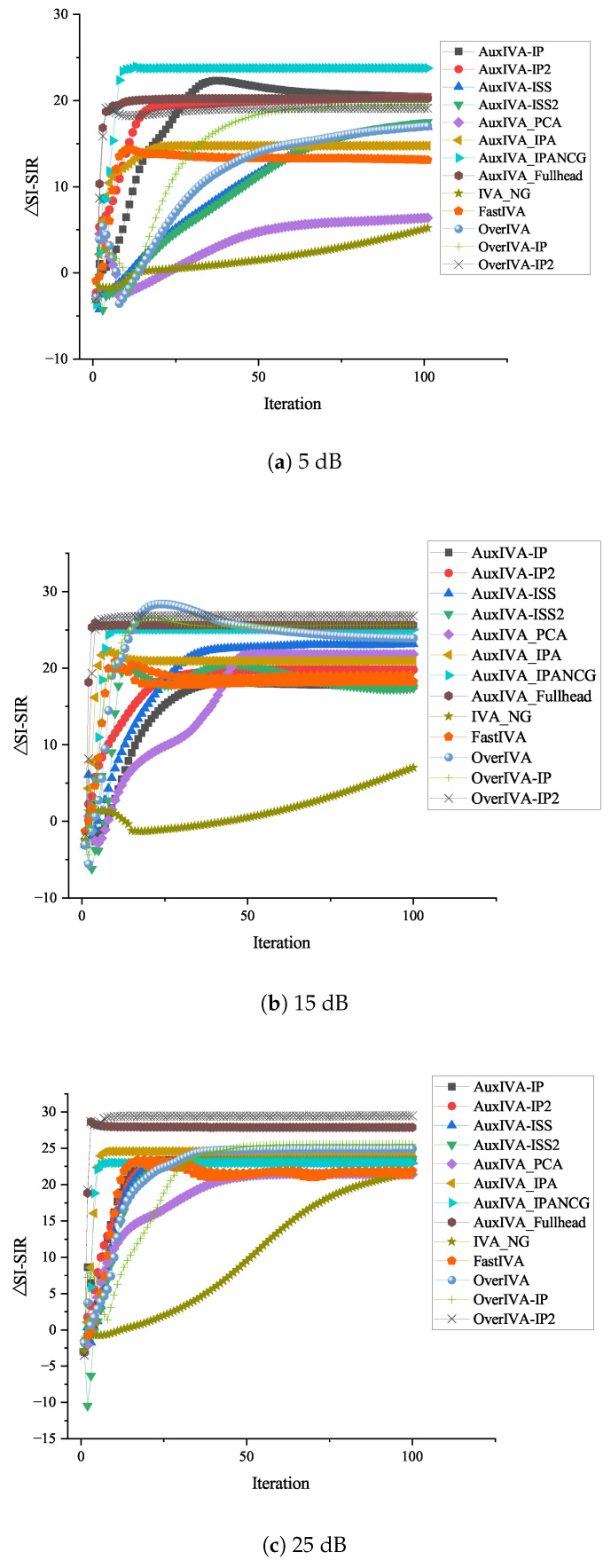
Performance comparison under different SNRs in the 4 × 3 overdetermined case.

Figure 11a–c shows three microphones and three target signals, and the SNR values are the performance comparison of the optimized update rule under the conditions of 5 dB, 15 dB, and 25 dB, respectively. Figure 12a–c shows four microphones and three target signals, and the SNR values are the performance comparison of the optimized update rule under the conditions of 5 dB, 15 dB, and 25 dB, respectively. Overall, it can be seen from the above performance comparison graphs that all methods optimized for (5) have similar distributions. Under the 3 × 3 model in Figure 11, AuxIVA-IPA and AuxIVA-IP2 have the best performance in a 5 dB environment, and AuxIVA-IP2 and AuxIVA-Fullhead have the best performance in a 15 dB environment. FastIVA outperforms other algorithms in the 25 dB environment, but the AuxIVA-IPA method has the most stable performance in the three cases and is the most favorable compared with the other algorithms. In the 4 × 3 model in Figure 12, the performance of the AuxIVA-IPANCG algorithm is significantly better than other algorithms in the 5 dB environment, and even surpasses the OverIVA method, probably because this method is more suitable for this specific scene. However, OverIVA-IP2 performs the best in the other two environments, indicating that this method is still the best choice when dealing with overdetermined models. In all cases, the NG method could not achieve convergence in the specified number of iterations; usually, more iterations were required to achieve convergence, and the method converged slowly. The IPA method jointly executes IP and ISS to update and updates one row and one column of the separation matrix in each iteration. At the same time, the method re-estimates the kth filter and adjusts the values of all other filters by taking steps consistent with the current estimate of source k, so the separation effect is better.

Table 2 shows the comparison of the running time of different algorithms when three sources are separated by 5 dB, where F is the extraction of a single source.

It can be seen from the table that the running times of ISS and ISS2 are close and short, and the time complexity is low. IVE processes a single source, so time complexity is minimal. The time complexity of OverIVA, IPANCG, and FastIVA is moderate. The time complexity of IP, IP2, IPA, and NG is high.

**Figure 13:**
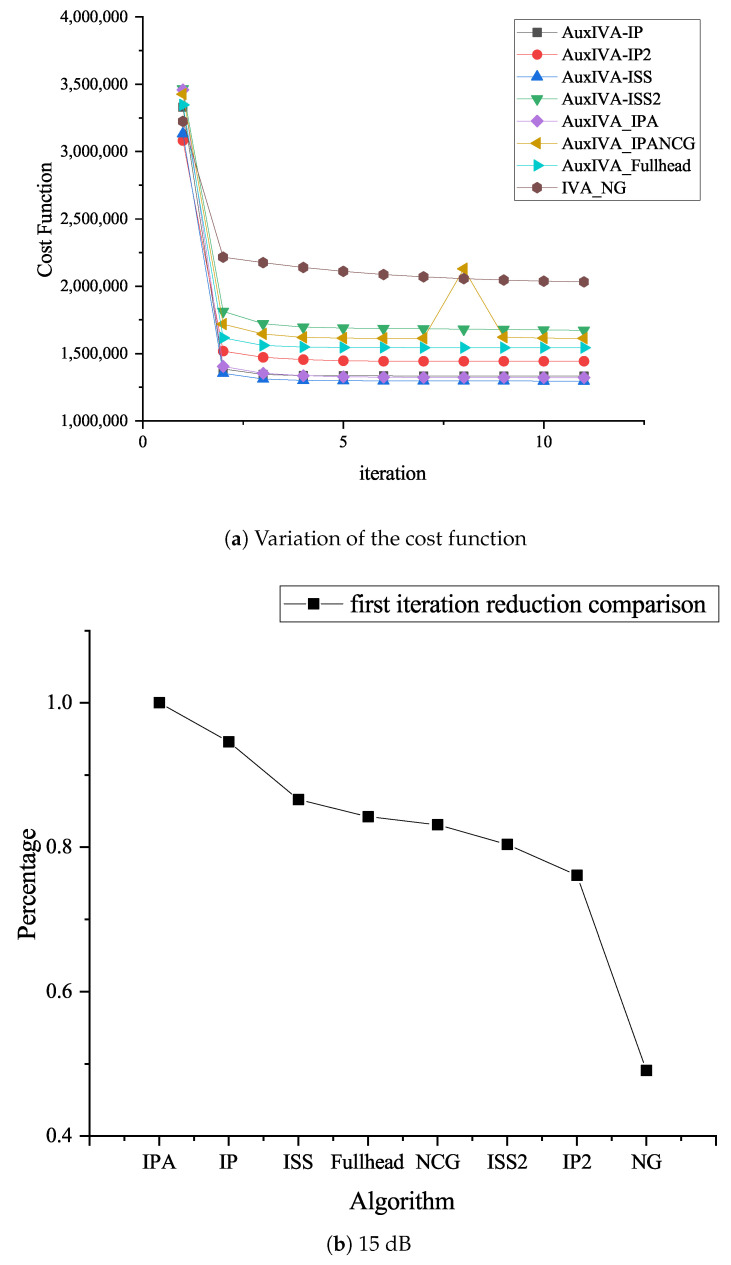
Cost function comparison at 5 dB.

**Figure 14:**
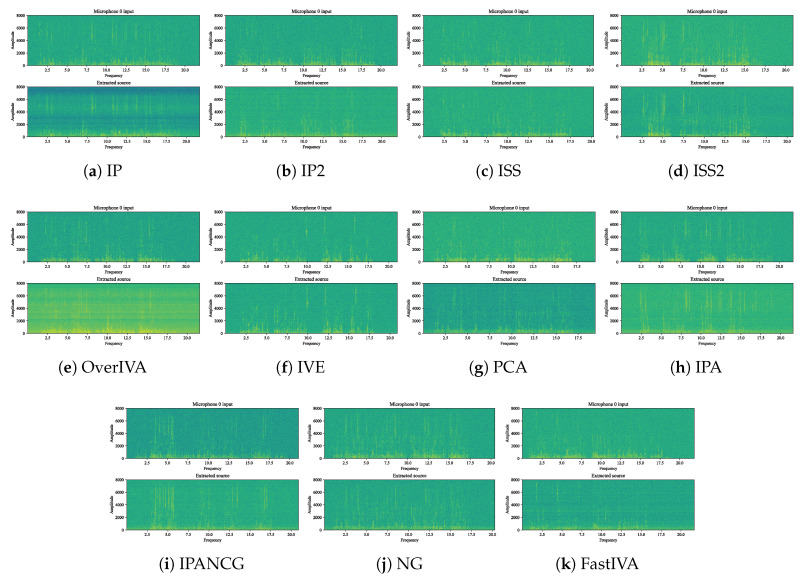
Separation effect comparison.

Figure 13a is a comparison of the cost function in a 5 dB environment. Figure 13b shows the reduction percentage of the cost function compared with AuxIVA-IPA after one iteration in the 5 dB environment. By comparing the two graphs, it can be concluded that the AuxIVA-IPA algorithm has the best performance in the 5 dB environment: the cost function declines the fastest, and the convergence speed is the fastest. Through the experiment, the specific signal separation effect diagram can be obtained as follows:

Figure 14 is the separation effect diagram of different methods in the 5 dB environment; OverIVA and IVE correspond to source separation and single-source extraction in the overdetermined environment, respectively. The remaining methods are used to determine source separation in the environment. From the separation effect, it can be seen that AuxIVA-IPANCG has a relatively good separation effect in a definite environment, OverIVA has a good separation effect in an overdetermined environment, and the IVE method has a very good effect on single-source extraction.

Through the above various numerical experiments, we can clearly understand the separation performance of different optimization update rules in the IVA algorithm in different scenarios. Through performance comparison, appropriate methods can be selected for source separation or source extraction in different scenarios. We note that the run results will be limited by software-based implementations and that more efficient implementations may be possible.

## 5. Summary and Prospect

In this paper, the optimization update rules of the principle of the IVA algorithm and the application of IVA in BSS are reviewed. The basic principles of the ICA and IVA algorithms are discussed. As an efficient method, the IVA algorithm can select appropriate optimization and update rules according to different separation scenarios. The optimization update rules based on IVA are mainly divided into six types: gradient method, Newton method, auxiliary function method, block coordinate method, expectation maximization, and eigenvalue decomposition. From the point of view of convergence speed and separation effect, the basic principles of these methods are briefly discussed. As the mainstream algorithm to solve the problem of BSS, IVA solves the problem of the ICA algorithm arrangement ambiguity and so on. Additionally, the source signal can be efficiently separated, and the improvement of the optimization update rule cannot only accelerate the convergence but also improve the overall separation effect. By reading the relevant literature, it is known that some of the above optimization update rules can be used interchangeably to speed up the entire convergence speed and achieve rapid separation. This is a thought-provoking and very interesting research direction, and related work will be carried out in the follow-up research.

This paper also conducts an experimental comparison of the existing optimization update rules. Through the numerical experiment comparison, the separation effect of the existing optimization schemes can be understood. In different scenarios, the corresponding update rules with good performance can be selected for blind separation work.

## Figures and Tables

**Table 1:** Optimize update rule classification.

Method	Principle	Characteristic	Step	Reference
NG	Step size selection mechanism for iterative update	The choice of step size affects the convergence; convergence speed is slow.	✓	[8,21,22,23,24,25,26,27,28,29,30,31]
FastIVA	Finding the optimal solution using Fast fixed point method	Faster convergence speed and low computational complexity.	✗	[32,33,34,35,36,37,38,39,40]
AuxIVA	Construct helper functions to estimate the unmixing matrix	Faster convergence speed, stability, and widely used	✗	[10,41,42,43,44,45,46,47,48,49]
EM	Estimate the parameters to calculate the expected value of the objective function	Handling scenarios where parameter estimation is complex or impossible	✗	[50,51,52,53,54,55,56,57,58]
BCD	Perform a linear search along a single dimension at a time, looping until convergence	Dealing with nonconvex functions that are difficult to obtain global optimum	✗	[42,59,60,61,62,63,64,65,66,67,68,69,70]
EVD	Eigenvalue decomposition	The mixing matrix is unmixed and updated by eigenvalue decomposition, and the update speed is fast	✗	[11,71]

**Table 2:** Algorithms’ running time.

	IP	IP2	ISS	ISS2	OverIVA	FIVE	IPA	IPANCG	NG	FastIVA
time(s)	14.455	14.347	13.291	13.357	13.912	7.884	14.481	13.718	14.657	13.71
